# Egypt's COVID-19 Recent Happenings and Perspectives: A Mini-Review

**DOI:** 10.3389/fpubh.2021.696082

**Published:** 2021-08-16

**Authors:** AbdulRahman A. Saied, Asmaa A. Metwally, Norah Abdullah Bazekh Madkhali, Shafiul Haque, Kuldeep Dhama

**Affiliations:** ^1^Department of Food Establishments Licensing (Aswan Branch), National Food Safety Authority (NFSA), Aswan, Egypt; ^2^Touristic Activities and Interior Offices Sector (Aswan Office), Ministry of Tourism and Antiquities, Aswan, Egypt; ^3^Department of Surgery, Anesthesiology, and Radiology, Faculty of Veterinary Medicine, Aswan University, Aswan, Egypt; ^4^College of Nursing, Jazan University, Jazan, Saudi Arabia; ^5^Research and Scientific Studies Unit, College of Nursing and Allied Health Sciences, Jazan University, Jazan, Saudi Arabia; ^6^Bursa Uludağ University, Faculty of Medicine, Bursa, Turkey; ^7^Division of Pathology, ICAR-Indian Veterinary Research Institute, Bareilly, India

**Keywords:** COVID-19, Egypt, case fatality rate, SARS-CoV-2, vaccines, medicinal plants, health care workers, mutations

## Abstract

The coronavirus disease 2019 (COVID-19) pandemic, caused by severe acute respiratory syndrome coronavirus 2 (SARS-CoV-2), has affected countries across the world. While the zoonotic aspects of SARS-CoV-2 are still under investigation, bats and pangolins are currently cited as the animal origin of the virus. Several types of vaccines against COVID-19 have been developed and are being used in vaccination drives across the world. A number of countries are experiencing second and third waves of the pandemic, which have claimed nearly four million lives out of the 180 million people infected globally as of June 2021. The emerging SARS-CoV-2 variants and mutants are posing high public health concerns owing to their rapid transmissibility, higher severity, and in some cases, ability to infect vaccinated people (vaccine breakthrough). Here in this mini-review, we specifically looked at the efforts and actions of the Egyptian government to slow down and control the spread of COVID-19. We also review the COVID-19 statistics in Egypt and the possible reasons behind the low prevalence and high case fatality rate (CFR%), comparing Egypt COVID-19 statistics with China (the epicenter of COVID-19 pandemic) and the USA, Brazil, India, Italy, and France (the first countries in which the numbers of patients infected with COVID-19). Additionally, we have summarized the SARS-CoV-2 variants, vaccines used in Egypt, and the use of medicinal plants as preventive and curative options.

## Introduction

**Figure 1:**
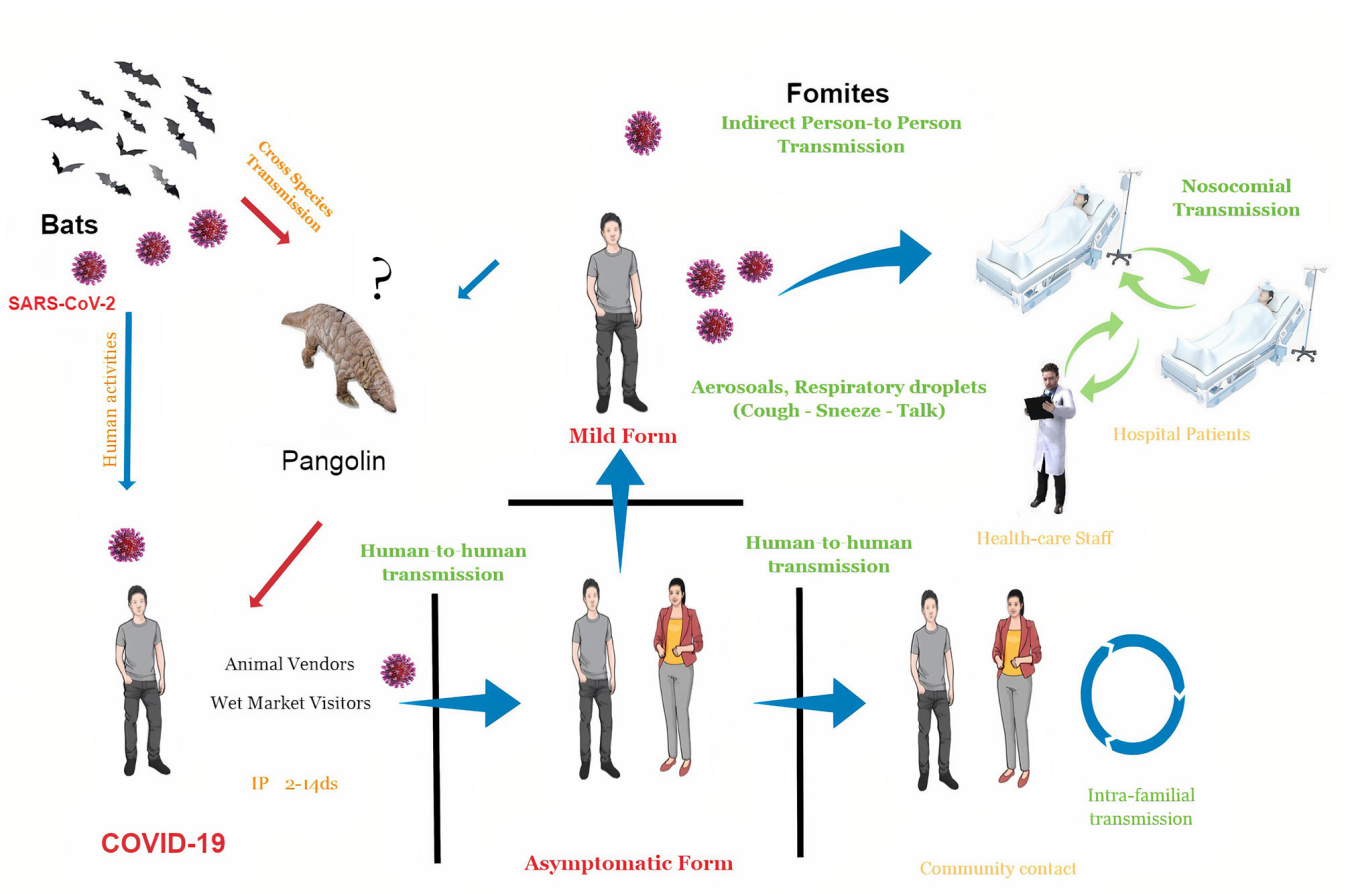
The schematic diagram of coronavirus disease 2019 (COVID-19) spread. Severe acute respiratory syndrome coronavirus 2 (SARS-CoV-2) is most likely spread from bats to people through intermediate hosts like pangolins or other wild animals sold at Huanan local Seafood Wholesale market, Wuhan, Hubei Province, China. Human-to-human transmission is established once the virus has been transferred in this manner. Nosocomial transmission (viral spread between persons inside hospitals, such as doctors, nurses, and patients) and intrafamilial transmission (viral spread between family members) are the most common kinds of human-to-human transmission.SARS-CoV-2 is transmitted through aerosol droplets and fomites. The disease has a 2- to 14-day incubation period and can cause mild, moderate, severe, or even asymptomatic forms.

**Figure 2:**
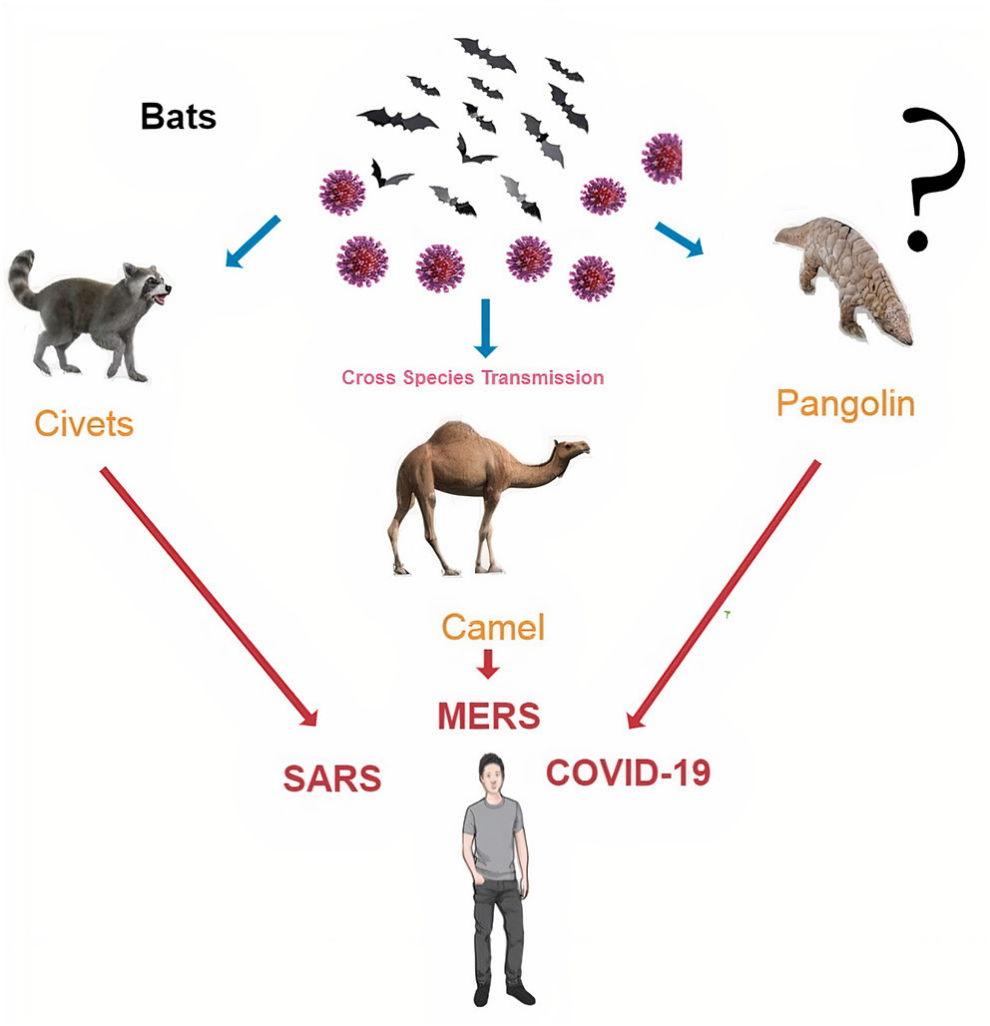
The last three coronavirus pandemics that human populations faced are SARS, Middle East respiratory syndrome (MERS), and COVID-19. They are originated in bats and then transmitted to humans *via* intermediate hosts, such as civet in SARS, dromedary camel in MERS, and pangolins mostly in COVID-19.

In late December 2019, a new coronavirus (SARS-CoV-2) emerged in Wuhan, Hubei Province, China (1). According to some reports, it first appeared on November 17, 2019 (2). Later on, severe acute respiratory syndrome coronavirus 2 (SARS-CoV-2) has widely and rapidly spread in China and several other countries, causing an outbreak of acute viral pneumonia and a pandemic disease called coronavirus disease 2019 (COVID-2019) (3, 4) (Figure 1). At the time of the writing of this review article, the SARS-CoV-2 has infected nearly 180 million and killed around 4 million (2.2% mortality rate) people worldwide. SARS-CoV-2 belongs to the genus *Betacoronavirus*

subgenus *Sarbecovirus*, subfamily *Orthocoronavirinae*, family *Coronaviridae*, and order *Nidovirales* (1, 5). SARS-CoV-2 binds to angiotensin-converting enzyme 2 (ACE-2) receptor in the body cells, primarily lung tissue *via* the S protein (the viral structural proteins, spike shape), which could be modified by transmembrane proteinase-serine 2 (TMPRSS2), facilitating the entry of viral particles into the cell (6). COVID-19 pandemic returns our minds to the ongoing ability of viral spillover from animals to cause severe disease in humans (1). Emerging zoonotic viruses continuously circulate in animal reservoirs, and events like cross-species jumping and zoonotic spillover might complicate the containment of viral pandemics. It is not new to say that SARS-CoV-2 will not be the last virus transmitted in this way. Coronaviruses (CoVs) are genetically diverse, undergo frequent mutations and recombinations (5, 7), and can jump from animals to humans, from humans to animals, and between animals (8, 9). The human genome evolves at a rate of 1% every 8 million years, whereas, many animal RNA viruses evolve at a rate of 1% every day, allowing us to predict the emergence of new zoonotic viruses (10). COVID-19 outbreak is thought to have begun in the Huanan local Seafood Wholesale market in Wuhan, Hubei province, central China (1). However, a very recent report indicated that the origin of COVID-19 might be from the wild animal farms that provided this market with animals (11). SARS-CoV-2 may be transmitted directly from bats (to sellers and market vendors) or utilizing intermediate animal hosts [e.g., pangolins (12) or other wild animals (13) to humans], and its zoonotic concerns are being investigated yet to reach any conclusion. SARS-CoV-2 has been detected in few animal species, such as dogs, cats, gorillas, tigers, lions, puma, and minks. Most of these instances have been linked to human-to-animal transmission; nevertheless, the virus was observed to spread rapidly among minks, with reports of mink-to-human transmission events (9, 14–16). Close human-animal contact and often coexistence are still common in rural African communities with virtually no barriers to wild environments (e.g., tropical forests), as they are in China. According to both the number of infected people and the geographic scope of the epidemic areas, COVID-19 has overwhelmingly surpassed SARS and Middle East respiratory syndrome (MERS) (17) (Table 1 and Figure 2).

**Table 1:** Severe acute respiratory syndrome (SARS), Middle East respiratory syndrome (MERS), and coronavirus disease 2019 (COVID-19).

**Disease**	**SARS**	**MERS**	**COVID-19**
Date of emergence	November, 2002	June, 2012	December, 2019
Origin	Guangdong, China	Jeddah, Saudi Arabia	Wuhan, China
Number of infected persons	8,422	2,499	135,471,700
CFR%	10.88% (916)	34.45% (861)	2.16% (2,931,916) as of Apr 9, 2021

Since mid-December 2019, human-to-human transmission has occurred among close contacts. SARS-CoV-2 is transmitted *via* the airborne route (spreads through the air) in indoor environments (18) and aerosol route (19) coming from either the breath, coughing, and sneezing of patients or aerosolization of the virus-laden aerosols from the feces or urine of a patient while using the toilet (20). Pathogen-bearing droplets of all sizes can travel 7–8 m (21). In addition, SARS-CoV-2 can remain stable for several days in aerosols and surfaces (18). The virus-laden droplets settle out or evaporate at rates that depend on their size, speed of the gas cloud, and the ambient environment properties (temperature, humidity, and airflow) (22). As the liquid content of droplets evaporates, several droplets become so small that they can pass through the air, bringing their viral content meters and tens of meters away from their source. The fecal-oral route is also reported as a route for SARS-CoV-2 transmission (23, 24) besides the fecal-aerosol transmission in a high-rise building in Guangzhou, China (25). The virus also can transmit from pregnant moms to their newborns *via* the transplacental and vertical routes (26, 27). COVID-19 causes asymptomatic infections and mild to severe pneumonia (28, 29). Its incubation period is 2–14 days (range from 2 to 7) (30), death period is 17–24 days.

Symptoms include fever, cough, dyspnea, muscle ache, confusion, headache, sore throat, rhinorrhea, chest pain, diarrhea, nausea, vomiting, anosmia, and dysgeusia (31). Acute respiratory distress syndrome (ARDS), cytokine storm, cardiovascular complications, pulmonary acute stroke, gastrointestinal and neurological manifestations, and kidney dysfunction are serious conditions that may lead to multiple organ failure and death (29, 32–34). Prevention and control measures include early diagnosis, contact tracing, strengthening of medical facilities, and adopting frequent handwashing, appropriate room ventilation, open space, social distancing, sanitization of protective apparel and proper use, disinfection of toilet areas, strict quarantine, travel restrictions, and minimizing the number of people sharing the same environment. To limit the danger of exposure to the airborne virus, public personal protection measures such as wearing masks, adopting social distancing rules, and avoiding crowded areas are critical (29, 35, 36).

To provide insight into the current scenario of the COVID-19 outbreak in Egypt, we used data from publicly accessible and up-to-date sources such as published papers, WHO reports, official newspapers of Egypt, and other reports.

## How Did Egypt Respond to Covid-19?

The following is a rundown of a timeline of main incidents that occurred recently in Egypt to slow down and control the spread of the virus: on January 26, 2020, all the flights between China and Egypt have been suspended, while the suspension of all flights began from March 19, 2020. Schools, universities, and all public areas where people gatherings could happen were closed. As of March 21, 2020, all mosques and churches were closed. Aside from that, external and internal tourism to tourist cities, including Luxor and Aswan, where cases have been confirmed among Egyptians and tourists onboard a floating hotel cruise, has been halted to prevent illness transmission. A curfew was enforced till the end of March 2020.

Campaigns of “Stay home, stay safe” for adopting social distancing on the social networks were performed extensively to create COVID-19 pandemic awareness among the public. Campaigns were launched in media and on the roads to promote frequent handwashing, cough etiquette, the use of personal protection equipment (e.g., facemasks), reducing hand-to-face contact, avoiding sharing bedrooms and towels, diminishing air conditioner (AC) use, and avoiding crowding in public transport. Also, the public was motivated to report fever and other symptoms, risk factors for coronavirus infection, including travel history to the affected areas, and close contacts with confirmed or suspected cases. The Egyptian Ministry of Health and Population (MOH) has launched a specialized hotline to provide medical counseling services for people in need. All festivals were suspended, and the number of employees was reduced in non-vital works to encourage public sector employees to work remotely, minimizing their contact and contamination of workplaces with the virus. The Egyptian government has carried out a massive disinfection program prioritizing all squares, workplaces, touristic locations, touristic hotels (permanent and floating), and restaurants, using chlorine-containing disinfectants as lipid solvents. Due to the economic impact of closure and curfew, all the previous restrictions were relaxed (37) to varying degrees and according to the epidemiological status, with tightening of movement restrictions on the largest religious festivities of 2020, such as during Eid Al-Fitr and Eid Al-Adha to avoid large gatherings and prevent SARS-CoV-2 infection. Egypt is a developing country, and tighter movement restrictions will undoubtedly have a short-term impact on its economy, particularly the tourism sector, a cornerstone of the economy of Egypt, which has been passed through difficult times since the 25 January revolution until the emergence of COVID-19 in Egypt. Therefore, Egypt became partially open, allowing for continued labor under more sanitary conditions, particularly in establishing large-scale projects. Thanks to the early measures implemented, Egypt could contain the rate of infection and minimize fatalities.

## Coronavirus Disease 2019 Statistics in Egypt

**Figure 3:**
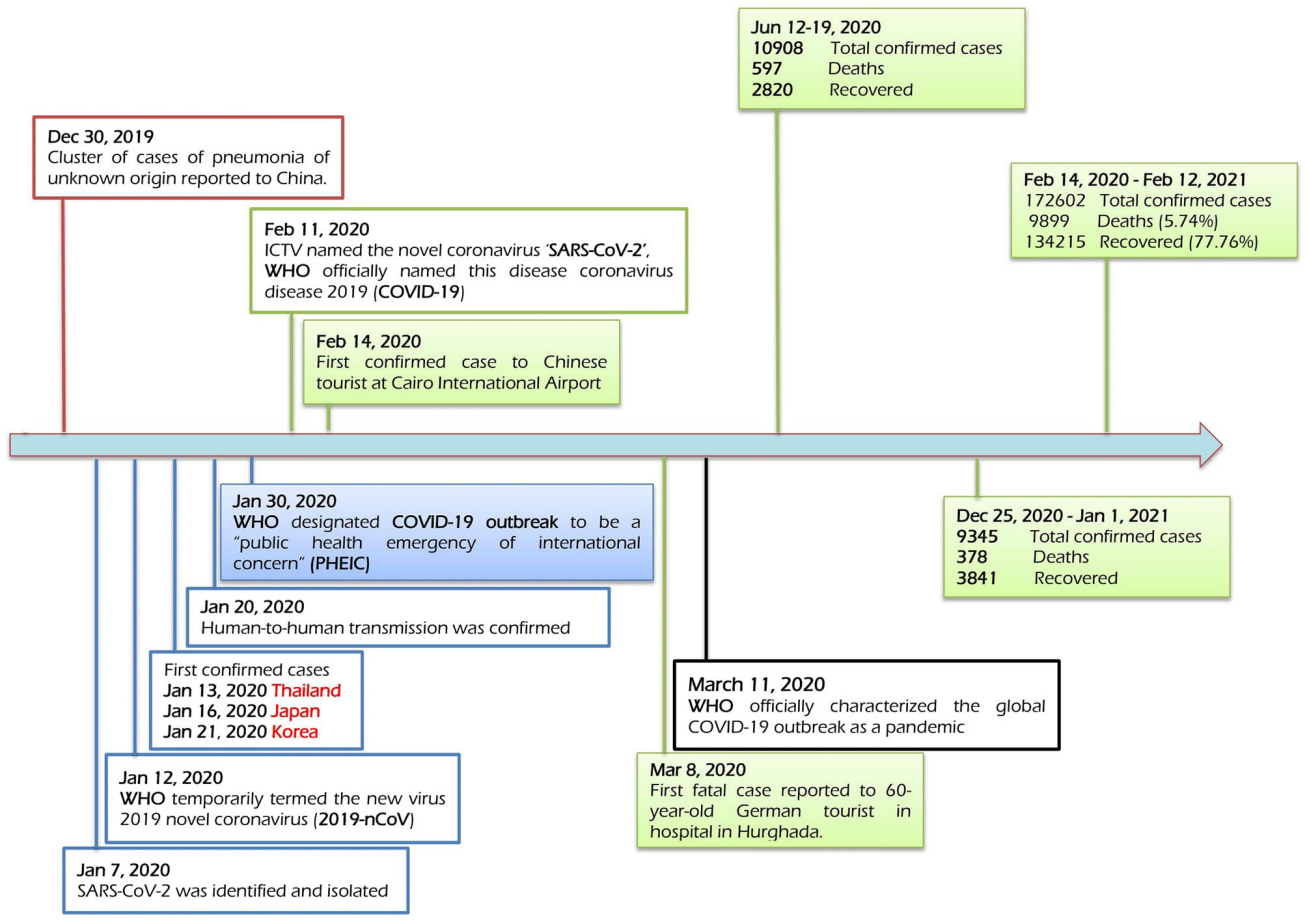
Timeline of the key events of the COVID-19 outbreak in Egypt. This timeline has recorded the events chronologically since the emergence of SARS-CoV-2 in China till the appearance of the first case in Egypt, forwarding till April 2021. It has recorded the first virus case (February 14, 2020) and the first death case (March 8, 2020). It has recorded the first and second waves of COVID-19 in Egypt in mid-June and late-December 2020, respectively.

**Figure 4:**
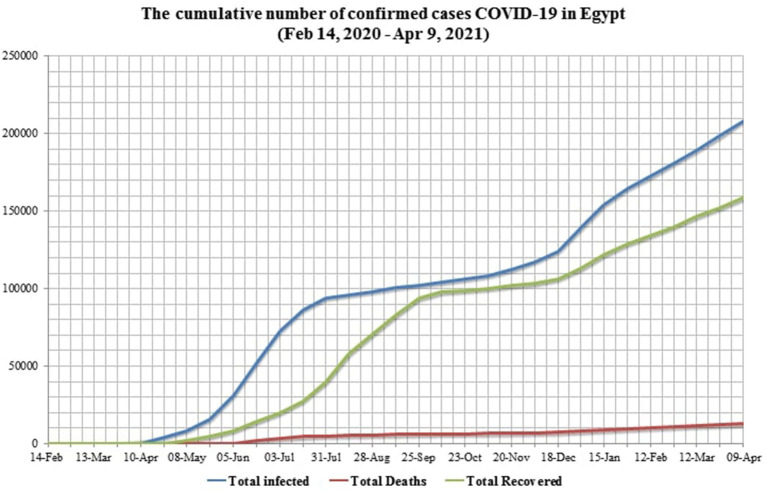
The chart shows the total confirmed cases in Egypt from the first confirmed case on February 14, 2020, till April 9, 2020. In Egypt, the first case of COVID-19 was confirmed on February 14, 2020, while the first death of COVID-19 was on March 8, 2020. Case fatality rate (CFR) reached 7.53% in early April 2020 and then afterward remained stable at around 5%. The percentage of recovered cases slowly increased till reaching 42.13% as of July 2020 and 91.14% as of late September. Recovered cases (RC%) reduced from November 2020 to April 2021 down to 75.24%. Active cases, on the other hand, increased from late December 2020 (10.63%) till early April 2021 (18.22%).

**Figure 5:**
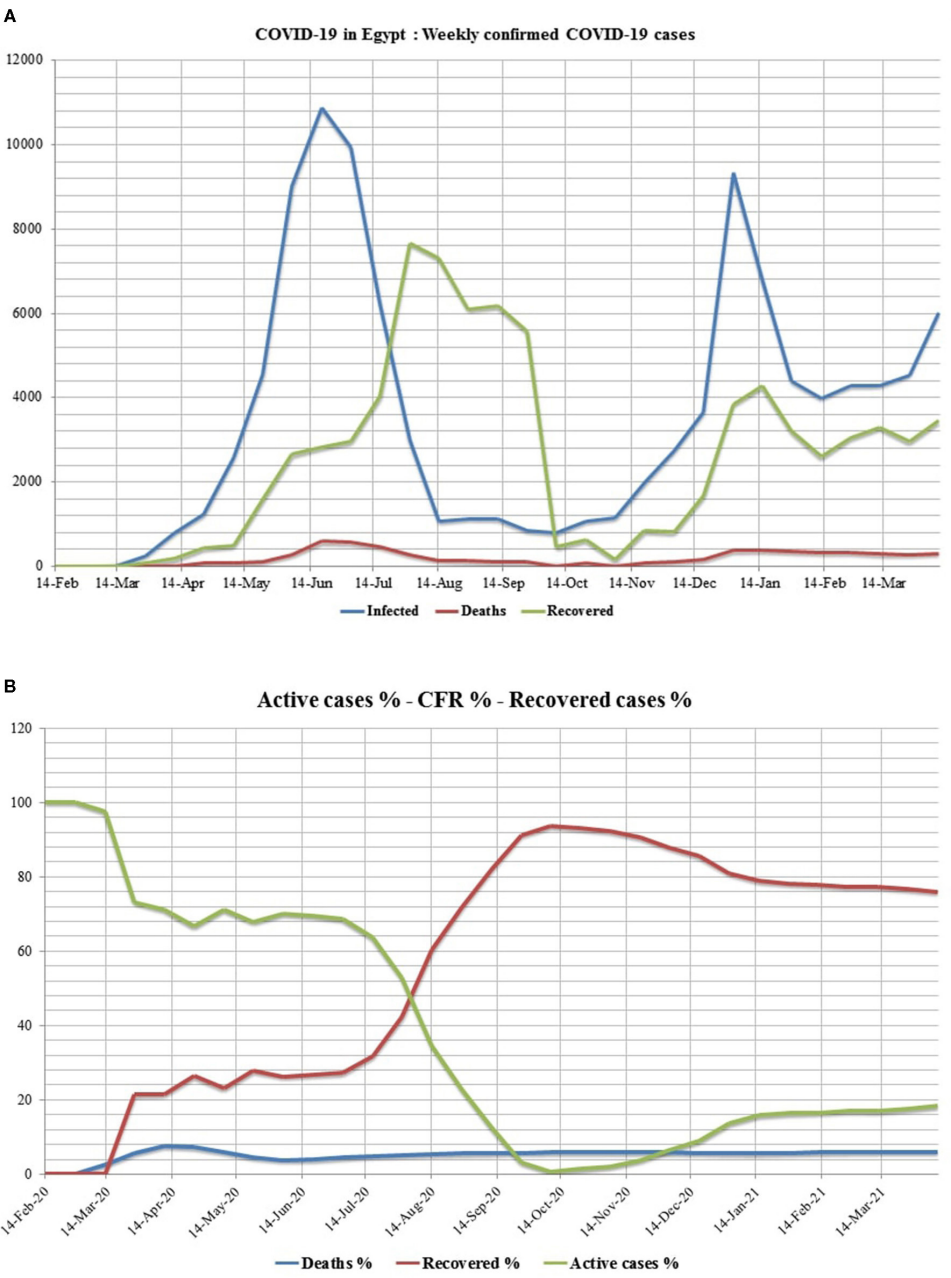
Chart shows **(A)** the weekly confirmed cases and **(B)** the weekly percentages of CFR, active cases, and recovery. The chart of the weekly confirmed cases showed the first and second waves in June and December 2020, respectively. The highest percentage of recovered cases was found in August 2020, while the lowest was found in November 2020. The death rate is correlated with the waves, but it was higher in the first wave. The charts of weekly percentages showed that the percentage of active cases started with the first confirmed case in Egypt (February 14, 2020) while the percentage of death rate started with the first death (8 March). The lowest percentage of active cases was recorded in October 2020, when the percentage of recovered cases was the highest.

Egypt confirmed its first case of COVID-19 on February 14, 2020, as the first African country had reported a confirmed case (Figure 3). From February 14, 2020, to April 9, 2021, 208,876 laboratory-confirmed infections, including 12,362 deaths (5.92%) by SARS-CoV-2 infection (Figure 4), were recorded according to the official website of the Egyptian MOH (https://www.care.gov.eg/EgyptCare/index.aspx accessed Apr 9, 2021) specialized for the news of COVID-19 outbreak in Egypt. Although, the incidence and morbidity rates are low, Egypt ranks the 7th country in case fatality rate (CFR 5.92%) with COVID-19 (Figures 5A,B). Egypt had high importation risk of SARS-CoV-2 cases from China (38), and by mid-March 2020, local transmission has been established (39). Genomes shared by Egypt with the GISAID^1^ are 957 and 0.343% of cases sequenced and shared (https://www.gisaid.org/index.php?id=208 accessed June 25, 2021). Some Egyptian genomic strains sequenced are similar to isolates from the USA, Austria, Sweden, Saudi Arabia, and France (40). In Egypt, the D614G variant^2^ is the most dominant (40–42). T851, F307F, 15907G, P323L, Q57H, Q57L, Q822K, V5F, G15S, T148I, G212V, K2798R, and T5020I are examples of mutations reported in Egypt (41). D614G and P4715L mutations are linked to transmissibility, regardless of symptom variability (43), while Nsp6-L3606fs, spike-glycoprotein-V6fs, and nsp13-S5398L variants may be linked to the worsening of clinical symptoms (43). The E3909G-nsp7 variant was shown to be more frequent in children and could explain why children recover so quickly (43).

In comparison with China (the epicenter of COVID-19 pandemic), the USA, Brazil, India, Italy, and France (the first countries in which the numbers of patients infected with COVID-19), Egypt ranks the 4th after China, India, the USA, and Brazil in terms of population. In terms of infected case percentage to population, Egypt ranks second-to-last after China, although, this does not reflect the whole picture of the epidemic in these countries. The COVID-19 recovery rate of Egypt is lower than China, India, Brazil, and Italy but similar to the United States. India and Egypt have the lowest mean age (28 and 25 years, respectively), and the majority of their people live in cities (65 and 57%, respectively). However, the CFR percentage in Egypt (5.92%) at the time of the writing of this article is higher than that of India (1.32%) (Table 2). It could reflect, in our opinion, that high numbers of infected cases in Egypt pass officially undetected and homely recovered (44) and the dependency of most Egyptian public on symptom-based diagnoses, such as anosmia and dysgeusia. On one hand, the low COVID-19 mortality rate in Africa is due to the lower average age of the population, shorter life expectancy, and a few cardiovascular disease patients (45). On the other hand, milder cases are undetected, and death is delayed, making it difficult to anticipate the case fatality risk accurately (46). The mortality risk appears to be significantly increased by age (47, 48) and comorbidities (cardiovascular diseases, cancers, diabetes mellitus, and chronic lung diseases) (49, 50). Patients with one or more comorbidities had a worse survival rate (51). People aged 46 years and above represented 91.5% of coronavirus-related deaths in Egypt (52). SARS-CoV-2 coinfections with other infectious agents have been reported in Egypt (Table 3).

**Table 2:** COVID-19 statistics in Egypt, Italy, France, India, Brazil, the USA, and China.

**Country**	**Egypt**	**Italy**	**France**	**India**	**Brazil**	**USA**	**China**
Population	102,334,404	60,461,826	65,273,511	1,380,004,385	212,559,417	331,002,651	1,439,323,776
Infected	203,546	3,650,247	4,741,759	12,476,468	12,912,379	31,319,713	101.773
	0.2%	6.04%	7.26%	0.9%	6.07%	9.46%	0.007%
Death	12,084	110,704	96,280	164,610	328,366	567,697	4,841
	5.94%	3.03%	2.03%	1.32%	2.54%	1.8%	4.76%
Recovered	155,448	2,974,688	297,734	11,625,318	11,276,628	23,826,158	96,570
	76.37%	81.49%	6.28%	93.18%	87.33	76.07%	94.89%
Land area (Km^2^)	995,450	294,140	547,557	2,973,190	8,358,140	9,147,420	9.388,211
Mean age (yrs)	25	47	42	28	33	38	38
Urban	43%	69%	82%	35%	88%	83%	61%
First case	Feb 14, 2020	Feb 20, 2020	Dec 27, 2019 Jan 24. 2020	Jan 30. 2020	Feb 25, 2020	Jan 23, 2020	Dec 8, 2019

*Data sources*.

*Worldometer. Population live updates (2020). Available at https://www.worldometers.info/coronavirus/ accessed on April 3, 2021*.

*Worldometer. COVID-19 coronavirus pandemic live updates (2020). Available at https://www.worldometers.info/population/ accessed on April 3, 2021*.

*https://www.worldometers.info/world-population/population-by-country/*.

*https://coronavirus.jhu.edu/map.html*.

**Table 3:** Reported coinfections with SARS-CoV-2 in Egypt.

Viruses	Influenza A (H1N1)	(44)
Bacteria	*Klebsiella pneumonia* *Acinetobacter baumannii* *Staphylococcus aureus* *Streptococcus pneumonia* *Enterococcus faecalis* *Escherichia coli* *Pseudomonas aeruginosa* *Enterobacter cloacae*	(53)
	*Legionella*	(54)
Fungal	*Candida albicans* *Candida glabrata*	(53)

Most COVID-19 deaths in Africa have been in older people (55) with non-communicable diseases. In the absence of effective control measures, regions with older populations may have disproportionately more COVID-19 cases, especially in the later phases of an unmitigated epidemic (47). Early and chronic pathogen exposure, which leads to persistent immune cell activation in harsh conditions, triggers a robust regulatory immune response to fight excessive inflammation (56). Monocytes from Africans who have been highly exposed to pathogens could be less pro-inflammatory (57). Immune antibodies that neutralize SARS-CoV-2, which were produced against earlier related human coronaviruses, may exist in people who have not been exposed to SARS-CoV-2 due to immunological cross-reactivity (58), which could explain decreased susceptibility (59, 60).

Dr. Hala Zayed, the Minister of Health and Population, admitted that the infection of Egyptians with the virus is much more than what the government has stated (61), and the same meanings were carried by the words of the Minister of Higher Education and Scientific Research of Egypt, Khaled Abdel Ghaffar (62), and that seems normal according to insufficiency closure and social distancing. In addition, numerous mild and asymptomatic patients might not receive a timely diagnosis or healthcare, free movement of these people [~40–45% (63)], such as presymptomatic travelers (64), helps in the spread of the virus to their contacts (fomites), concealing the true incidence and allowing disease progression. Therefore, asymptomatic people could not be diagnosed in January 2020, contributing to the spread of the epidemic. The viral shedding in the asymptomatic patients and survivors (65, 66) lasts for about 20 days, and this was significantly longer than that in the symptomatic patients (65). Generally, 81% of COVID-19 cases are asymptomatic and have mild disease, while severe cases are 14%, and critical and deceased cases are 5% (17). Kissler et al. (59) suggested a short duration of immunity after SARS-CoV-2 infection, while Long et al. (65) suggested that asymptomatic individuals had a weaker immune response to SARS-CoV-2 infection. Lack of severe disease manifestations in clinically healthy people infected with SARS-CoV-2 could pose a significant risk to vulnerable populations with underlying medical conditions (diabetes, hypertension, or cardiovascular disease) (24, 67, 68), proposing that the outbreak has several peaks.

Cruise ships carry different people into close contact for many days, making it easier for respiratory illnesses to spread (69). At least 60 laboratory-confirmed cases in the USA were linked to Nile River cruises in Egypt since February (70). Additionally, COVID-19 cases from Taiwan, UAE, France, and Japan were linked to travels from Egypt (71, 72). All the reported cases abroad are not within the estimates of the Egyptian government (73). El Gouna film festival was held in EL Gouna city from October 23, 2020, to October 31, 2020, which witnesses many Egyptian stars being infected with SARS-CoV-2. Later on, the curve of SARS-CoV-2 infection exponentially increased to the second wave and probably that the patient tracing would not be efficiently performed. Recent reports (74) reported a slow and continual increase in infections and deaths in Sohag after a deadly train crash (75) in early April 2021. It is attributed to the large numbers of people who were present in Sohag on the same day of the collision, such as officials, relatives of the injured, and ordinary people who gathered around the crashed trains to rescuing the people.

*"Fear doesn't prevent death. It prevents life,”* said Naguib Mahfouz. Egyptians are often affected by social distancing rules, as social gatherings and festivals are deeply ingrained in their culture. These include religious events, such as naming ceremonies, weddings, and communal prayers, especially on Fridays held at mosques and churches. According to the Pew Research Center, 62% of Egyptians attend worship services weekly, and 72% say religion is very significant to their lives (39). Since the majority of citizens work in the informal business sector, such as traditional markets, strict lockout policies are difficult to enforce (37). Although, the critics were directed to the inability of the Egyptian government to provide the basic protective equipment to healthcare workers (HCWs), Egypt successfully hosted the 2021 IHF^3^ World Men's Handball Championship, the first to involve 32 competing teams, from January 13, 2021, to January 31, 2021, without audience participation. The success of hosting this tournament is due to its launching behind doors besides releasing the Egypt 2021 COVID-19 Medical Precaution Plan.

## Egyptian Healthcare Workers

Personal protective equipment (PPE) kit, surgical masks, N95 masks, and goggles are examples of personal protective control measures, especially for healthcare providers treating infected patients (76). Although, the efforts were carried out to combat the pandemic, inadequate PPEs (77) had a deleterious effect on the life (78) and mental health (79) of Egyptian Healthcare workers (EHCWs). According to the Egyptian Medical Syndicate (EMS), around 400 doctors have died of the SARS-CoV-2 virus infection, despite the failure of the MOH to divulge information on the number of medical professionals who died of COVID-19, including physicians, nurses, and technicians (80) (Table 4).

**Table 4:** A total of Egyptian physicians who had contracted or died from COVID-19 infections by mid-October 2020.

**Egyptian physicians**		
Total Infected Cases	3,575	1.625% of total Egyptian physicians (220,000)
		3.4% of total confirmed cases (105,159)
Total Dead Cases	188	5.26% of total Egyptian physicians (3,575)
		3.08% of total confirmed cases (6,099)

*Data source*.

*Alarabiya News. Alarabiya News, 19 October. Syndicate: 3576 cases and 188 deaths of Corona among Egyptian physicians. Alarabiya News; 2020. (accessed 07 November 2020)*

*https://www.alarabiya.net/ar/arab-and-world/egypt/2020/10/19/. (Alarabiya News, 2020)*.

*Worldometer. COVID-19 coronavirus pandemic live updates (2020). Available at https://www.worldometers.info/population/ Accessed on April 3, 2021*.

A recent study showed that 68.2% of SARS-CoV-2 infections in EHCWs are asymptomatic infections, and most infected EHCWs were nurses (81). Another study found that workers responsible for transportation of patients and cleaning, nurses, and administrative employees were more likely to get SARS-CoV-2 infections higher than physicians. Moreover, 14.3% of frontline HCWs in the emergency department (ED) had contracted SARS-CoV-2 infections. This highlights the importance of more stringent infection control measures, education, and supervision to these HCWs alongside regular molecular testing of HCWs, even in the absence of symptoms, to protect HCWs from COVID-19 and reduce transmission from infected HCWs to the public (82, 83); that is why the Egyptian government has prioritized COVID-19 vaccination for HCWs and the elderly. A measles vaccine trial has been registered to prevent COVID-19 among HCWs in Egypt (84).

## Therapeutics and Vaccines

Recently, exploring several vaccine platforms and advances in research studies has paved the way for developing COVID-19 vaccines. Few of the approved vaccines are being used presently for vaccinating people in many countries, while other modern vaccines are in the pipeline of clinical trials and being conducted at various stages of development (85–89). However, there are currently no successful COVID-19 therapies or antivirals available for SARS-CoV-2. Various drugs and therapies, such as remdesivir, ivermectin, dexamethasone, convalescent plasma therapy, antibody-based immunotherapies, monoclonal antibodies, immunomodulatory agents, and others, have been found effective and utilized in an emergency to alleviate disease severity in COVID-19 patients; however, the choice of drugs and medicines are still been identified (3, 90–93) (Table 5). The use of antimalarial medications to treat malaria, notably artemisinin-based combination therapy (ACT), is one of the possibilities that could explain the later onset and spread of the COVID-19 pandemic in African countries, such as Egypt (95).

**Table 5:** Egyptian protocol for COVID-19 treatment/management.

**Antipyretic**	**Paracetamol**
**Cough suppressants**	**Acelylcysteine**
**Anticoagulants**	**Enoxaparine**
**Fluid therapy**	**According to the condition of the patient**
**Multivitamins**	**Vitamin C or Zinc**
**Antiviral drugs and Antibiotics**	**Hydroxychloroquine - Ivermectin – Favipiravir - Remdesivir - Lopinavir/Ritonavir - Monoclonal antibodies - Convalescent plasma - Azithromycin - Nitazoxanide- Oseltamivir - Ribavirin - Interferon beta 1b - Doxycycline**
**Anti-inflammatory**	**Hydrocortisone – Dexamethasone - Methylprednisolone**
**Supplement**	**Lactoferrin**
**Immunosuppressive**	**Tocilizumab**
**Oxygen therapy**	
**Mechanical ventilation**	

*For patients with COVID-19, the Egyptian Ministry of Health approved a standard of a care treatment strategy that included (94)*.

*By the end of May 2020, the Egyptian MOH guideline recommended that mild and some moderate cases will be managed by home isolation (53)*.

During the ongoing pandemic, benefits of balanced nutrition, dietary supplements, nutraceuticals, plant extracts, medicinal herbs, traditional medicines, and other alternative/supportive regimens have also gained momentum owing to their promising ability to boost immunity and to promote better health and possessing antiviral properties and, therefore, have revealed a potential role in managing and treating patients infected with COVID-19 (96–101). Alternative and supportive approaches to prevent and control the spread of the pandemic disease are in urgent need. In Egypt, where a large population has cultural and religious perceptions, using natural substances as immunomodulators and medicines is considered an old approach. In the case of respiratory viral infections, certain natural products with immunostimulatory and antiviral properties are recommended. These may be used as a treatment and a preventative measure against viral infection and replication. Several traditional herbal medicines have been confirmed to have antiviral properties against the SARS-CoV-2 (97–102) (Table 6). Licorice salesman is a profession that is extensively located in Egypt alongside Egyptian spices dealers.

**Table 6:** Summary of some functional food plants of anti-inflammatory, immunomodulatory, and antiviral activities that could have anti-SARS-Co-2 activity.

**Natural products**	**References**
*Artemisiaannua* (Sweet wormwood)	(102, 103)
*Broussonetia papyrifera* (Paper mulberry)	(104)
*Ocimum basilicum* (Basil)	(105)
*Mentha piperita* L. (Mentha)	(106, 107)
*Zingiber officinalis* (Ginger)	(108–110)
*Curcuma longa* (Turmeric)	(109–114)
*Ficus carica* (Fig)	(109)
*Allium sativum* (Garlic)	(107, 109)
*Glycine max* (Soybean)	(115)
*Citrus aurantium* (hesperidin and neohesperidin)	(116)
*Camellia sinensis* (Tea)	(109, 116)
*Oleanolic acid*	(116)
*Trigonellafoenum-graecum* (Fenugreek)	(107)
*Pelargonium sidoides*	(103)
*Isatis indigotica* (Woad)	(103)
*Glycyrrhiz aglabra* (Liquorice)	(103, 107, 109, 117–119)
*Punica granatum* L. (Pomegranate)	(109)
*Piper nigrum* L. (Black pepper)	(109)
*Citrus aurantium* L. (Bitter orange)	(109)
*Mangifera indica* L. (Mango)	(109)
*Psidium guajava* L (Guava)	(109)

We suggest that the anti-inflammatory activities of some previous medicinal plants may be responsible for the improvement of some infected patients before lung tissue damage. Indomethacin, a non-steroidal anti-inflammatory drug, demonstrated potent antiviral activity against SARS-CoV-2 when used in combination with herbal preparations (120). In a recent study, indomethacin and resveratrol showed promising efficacy against SARS-CoV-2 (120–122). In Egypt, indomethacin is widely used in the treatment of many rheumatic conditions (123). The African Programme for Onchocerciasis Control (APOC) includes a group of African countries that had participated in an ivermectin campaign from 1995 to 2015 to combat onchocerciasis (124). Although, Egypt is a non-APOC country, the extra-label and extensive uses of ivermectin in the veterinary field in food-producing animals could have a role in the low incidence, but this speculation needs to be confirmed. Since December 2020, Egypt began to receive shipments of anti-COVID-19 vaccines, such as Sinopharm (BBIBP-CorV), AstraZeneca vaccine, and Sputnik V. Priority groups for vaccination are (A) medical staff at quarantine, fever, and chest hospitals, (B) patients with cancer or kidney or immunity problems, patients with chronic diseases, and the elderly, and (C) eventually all citizens above 18 years. As of March 2021, Egypt has started COVID-19 vaccine rollouts (125). Although, the country aims to vaccinate 40% of its population against COVID-19 by the end of 2021, Egypt is one of the countries that suffer from vaccine hesitancy due to misinformation and false claims about vaccines used in Egypt (126). In February 2021, the first private post-COVID-19 clinic was established in Cairo to assist in the treatment of post-acute sequelae of SARS-CoV-2 infection (PASC) in patients who had not previously been hospitalized with COVID-19 (127). Post-COVID-19 complications and long-term consequences have primarily affected older patients (over 65 years of age) with comorbidities (127).

## Limitations of This Study

Although, this review article depended on the laboratory-confirmed cases, many Egyptian people have caught SARS-CoV-2 infection and symptom-based diagnoses and homely isolated till recovery or hospitalized after the appearance of complications.

## Conclusions

Low COVID-19 prevalence in Egypt does not reflect the reality. Many SARS-CoV-2-infected people pass without any laboratory confirmations, and some cases depend on CT scans in informal laboratories. The wide traditional use of medicinal plants as hot or cold beverages and flavors added to foods could play a role in mitigating COVID-19 symptoms in Egyptians. The lower fatality rate in relation to the total population in Egypt (0.2%) and India (0.9%) might be due to the famous use of plants as prescriptions in both countries besides drinking of milk of cows, which may contain antibodies against this virus (128, 129). The lower incidence in Egypt may be attributed to the huge populations, low amount of screening testing, temperature and humidity, Bacille Calmette-Guerin (BCG) vaccine use (130), host genetics (131), misdiagnosis, and the young age of populations (132). Genetic variation has also been proven to alter the resistance of indigenous Africans to a variety of infectious diseases (133). The limitations of this study could make these findings a platform for adding insights to the existing knowledge. When living creatures put humanity in a tough problem across scientific history, other living creatures carry the solution for the problem. The interrelationship between human-animal-environment, including microbes and toxicants, is a very complicated issue that has not been clearly understood till now. Human behavior against animals and the environment and even other humans constitutes the main factor in this interrelationship. According to many experts, human overpopulation is the worst environmental problem in the world (134). In some countries, the implementation of strict restrictions to control the outbreak is difficult and could be impossible. So, we need to turn our eyes toward boosting our immunity and improve our behaviors toward the environment. Surveillance and monitoring of various animal species for SARS-CoV-2 and integrating one health approach need to be strengthened and be widely implemented along with ramping up of the global vaccination drive, prevention, control, and mitigation strategies to halt the ongoing pandemic. The extensive attention given to the animal viruses alongside altered human behaviors, environmental changes, and adequate strengthening of global public health facilities (135–137) could block a future global viral pandemic.

## Author Contributions

AS: conceptualization, methodology, formal analysis, investigation, validation, writing - original draft, and writing - review and editing. AM: writing - original draft, and writing - review and editing. KD, SH, and NM: funding acquisition and writing - review and editing. All authors agreed to the published version of the manuscript.

## Conflict of Interest

The authors declare that the research was conducted in the absence of any commercial or financial relationships that could be construed as a potential conflict of interest.

## Publisher's Note

All claims expressed in this article are solely those of the authors and do not necessarily represent those of their affiliated organizations, or those of the publisher, the editors and the reviewers. Any product that may be evaluated in this article, or claim that may be made by its manufacturer, is not guaranteed or endorsed by the publisher.

## Footnotes

^1^Global initiative on sharing all influenza data.

^2^The replacement of aspartic acid by glycine at position 614 of the spike glycoprotein, which dominated the sequence.

^3^International Handball Federation.
